# Contextual Modulation of Physiological and Psychological Responses Triggered by Emotional Stimuli

**DOI:** 10.3389/fpsyg.2013.00212

**Published:** 2013-05-10

**Authors:** Tomomi Fujimura, Kentaro Katahira, Kazuo Okanoya

**Affiliations:** ^1^Okanoya Emotional Information Project, Exploratory Research for Advanced Technology, Japan Science and Technology AgencyWako, Saitama, Japan; ^2^Emotional Information Joint Research Laboratory, Brain Science Institute, RIKENWako, Saitama, Japan; ^3^Center for Evolutionary Cognitive Sciences, The University of TokyoMeguro, Tokyo, Japan; ^4^Department of Life Sciences, Graduate School of Arts and Sciences, The University of TokyoMeguro, Tokyo, Japan

**Keywords:** emotional context, IAPS, skin conductance response, heart rate, facial electromyography

## Abstract

A series of emotional events successively occur in temporal context. The present study investigated how physiological and psychological responses are modulated by emotional context. Skin conductance response (SCR), heart rate, corrugator activity, zygomatic activity, and subjective feelings during emotional picture viewing were measured. To create an emotional context, a unpleasant or pleasant picture was preceded by three types of pictures, i.e., unpleasant, pleasant, and neutral pictures, resulting in six pairings. The results showed that viewing an unpleasant picture attenuated pleasant feelings induced by the following pleasant picture. On the other hand, preceding pleasant pictures decreased SCR to the following pictures. The effects of contextual modulation on emotional responses might be due to the informative function of pre-existing feelings; unpleasant feelings signal a threatening environment, whereas pleasant feelings signal a benign environment. With respect to facial muscle activities, viewing a pleasant picture decreased corrugator activity in response to the preceding picture. These findings suggest several types of contextual modulation effects on psychological, autonomic, and somatic responses to emotional stimuli.

## Introduction

Emotions involve several physiological and psychological changes. Many previous studies have found that viewing emotional pictures activates cardiovascular and electrodermal responses and the somatic system (Lang et al., 1993; Bradley et al., 1996, 2001; Bernat et al., 2006). For example, skin conductance responses (SCRs) are greater when viewing emotionally arousing pictures than when viewing neutral pictures. On the other hand, heart rate changes and facial muscle activity are predominately sensitive to the hedonic valence of pictures (Lang et al., 1993; Bradley et al., 2001). Heart rate deceleration is greater when viewing unpleasant pictures than when viewing pleasant or neutral pictures. As for facial activity, reactions of the zygomatic major muscle are facilitated by pleasant pictures, and reactions of the corrugator muscle are facilitated by unpleasant pictures. These physiological responses to pictures covary with psychological ratings of valence or arousal (Lang et al., 1993). Thus, it is obvious that the fundamental factors of emotion, valence, and arousal elicit physiological and psychological responses.

These emotional responses are fundamentally organized by two motivational systems, the defensive and appetitive systems (Lang et al., 1997; Bradley et al., 2001). The defensive system is activated by stimuli that threaten survival and results in facilitation of withdrawal, escape, and attack behaviors. The appetitive system is activated by stimuli that promote survival and procreation and results in facilitation of ingestion, copulation, and caregiving behaviors. Regarding to the emotional properties, the valence properties of a stimulus indicate whether the defensive or appetitive motivational system is active, and the arousal properties of a stimulus determine the intensity of the activation of the motivational system (Lang et al., 1990; Bradley et al., 2001). Given that these systems evoke the autonomic and somatic physiological responses to meet situational demands, whether an encountered stimulus is good or bad for an organism’s survival (i.e., the valence) and the extent to which the stimuli is significant (i.e., the arousal) might determine physiological and psychological patterns.

People can quickly classify perceived stimuli as “good” and “positive” or “bad” and “negative” (Fazio et al., 1986; Bargh et al., 1992). However, judgments of perceived stimuli can be influenced by pre-existing feelings. For example, you may reject a worthwhile proposal from your colleague if you have been irritated due to an earlier event. Such a phenomenon can be explained in a psychological framework termed the feelings-as-information hypothesis (Schwarz and Clore, 1983, 2007); according to this hypothesis, feelings related to affective, cognitive, and somatic experiences serve as information in the formation of evaluative judgments. That is, these experiences inform us about the current situation we are facing; pleasant feelings signal a benign situation, whereas unpleasant feelings signal a threat or problematic situation. From the feelings-as-information perspective, the motivational significance of a current emotional stimulus can be altered according to pre-existing feelings. In other words, the motivational significance of an emotional stimulus is not always absolute but depends on contexts involving other emotional stimuli. Taken together, it is possible that physiological and psychological responses to an emotional stimulus are modulated by other preceding emotional stimuli.

Regarding contextual modulation of emotional responses, there is an evidence that the valence properties of preceding stimuli are assimilated into the emotional responses to target stimuli. A previous study measured physiological and psychological responses when viewing emotional pictures preceded by emotional pictures with same or opposite valence (Waugh et al., 2011). The results indicated that self-reported affect in response to the current pleasant pictures was less pleasant when the previous picture set was unpleasant than when it was pleasant. Furthermore, corrugator electromyogram (EMG) activity was greater for the current unpleasant picture when the previous picture set was unpleasant than when it was pleasant. These results suggest that the valence properties of preceding stimuli are carried over into the emotional responses in terms of subjective feelings and facial activities elicited by target stimuli. From the feelings-as-information perspective, it is possible that feelings induced by pre-viewing of emotional pictures functions as information in the evaluation of the emotional significance of target pictures and results in modulation of the emotional responses to target pictures.

However, there are two issues about contextual modulation of emotional responses. One issue is that it is unclear whether the unpleasant or pleasant feelings induced by preceding stimuli impact emotional responses to the current stimulus because preceding neutral stimuli were not presented as a control condition in the previous study (Waugh et al., 2011). To clarify the emotional impact of preceding stimuli on target stimuli, the present study tested emotional responses to stimuli preceded by neutral, pleasant, and unpleasant stimuli. The other issue is that the effects of emotional context on the modulation of the arousal level of the motivational system in response to target stimuli have not been revealed. Waugh et al. (2011) found that emotional context only modulated subjective ratings of valence and corrugator activity, which are indices of valence. The present study indexed arousal with subjective ratings of arousal and SCRs. According to the feelings-as-information hypothesis, we expected that target stimuli preceded by pleasant stimuli would elicit less arousal than target stimuli preceded by neutral and unpleasant stimuli due to pre-existing pleasant feelings that signal a safe environment. On the other hand, we expected that target stimuli preceded by unpleasant stimuli would elicit more arousal than those preceded by neutral or pleasant stimuli because unpleasant stimuli signal a threatening environment. To allow pre-existing pleasant feelings to signal a safe environment, we used moderately arousing pictures as preceding pleasant stimuli. The set of pleasant pictures in this study included pictures of puppies, smiling children, and beautiful scenes. Furthermore, we selected unpleasant pictures that produced the same level of arousal as the pleasant pictures.

Thus, the present study investigated whether emotional context (i.e., a preceding emotional stimulus) modulated physiological and psychological responses to a current emotional stimulus using the picture-viewing paradigm. We hypothesized that, if the pleasant feelings induced by viewing pleasant pictures signal a benign environment, the target stimuli would elicit more pleasant and less aroused states. On the other hand, if unpleasant feelings induced by viewing unpleasant pictures signal a threatening environment, target stimuli should elicit more unpleasant and more aroused states. These modulations could be reflected in the following physiological and psychological responses: SCR, EMG activities, heart rate, and subjective valence and arousal ratings. The present study seeks to provide beneficial evidence about emotional responses in temporal contexts that are likely to occur in the real world.

## Materials and Methods

### Participants

Forty-three adults (24 men and 19 women; mean ± SD; 25.02 ± 6.74 years) participated in the study. The participants were recruited by advertisements placed with by intermediary company, and their occupational backgrounds varied widely. Participants received compensation for participating in the experiment. All participants had normal or corrected-to-normal vision. As some physiological data were missing because of equipment failure, one or more participants for each dependent measure were excluded from the analyses. The final sample sizes for each analysis were as follows: SCR, *n* = 43; corrugator activity, *n* = 40; zygomatic activity, *n* = 40; and heart rate, *n* = 40. All experimental procedures were approved by the Ethics Committee of the Japan Science and Technology Agency.

### Stimulus material and experimental design

The stimuli were 25 pictures selected from the International Affective Picture System (IAPS: Lang et al., 2008) that were composed of 10 unpleasant, 10 pleasant, and 5 neutral pictures^1^. The mean pleasantness/arousal ratings were as follows: 7.63/5.10 for the pleasant pictures, 4.99/2.45 for neutral pictures, and 2.73/5.23 for unpleasant pictures. All pictures were presented in full color.

**Figure 1:**
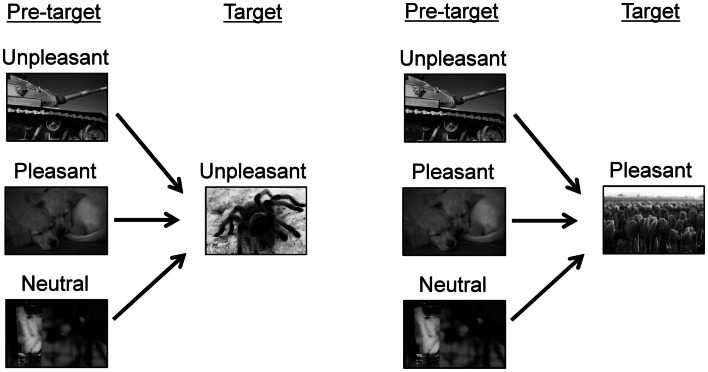
**Experimental conditions**. The sample pictures were taken from other source materials to avoid showing IAPS pictures in this paper.

The experimental conditions are shown in Figure 1. For each trial, two pictures were consecutively displayed. A pleasant or unpleasant target picture was preceded by one of three types of pictures, i.e., unpleasant, pleasant, or neutral pictures. Thus, six pairings were produced (i.e., unpleasant/unpleasant, pleasant/unpleasant, neutral/unpleasant, unpleasant/pleasant, pleasant/pleasant, and neutral/pleasant). These pairings were arranged into three blocks according to the hedonic content of the pre-target picture (unpleasant, pleasant, and neutral blocks). Each pre-target picture was presented two times within a block. In each block, 10 pairings of pictures were randomly presented, and these pairings including five unpleasant and five pleasant pictures as target pictures. The target pictures were identical across the three blocks. The order of the three blocks was randomized across participants.

### Apparatus and physiological measurement

Experimental events were controlled by a program written in Inquisit 3.0 (Millisecond) and were implemented on a computer (Vostro 420, Dell) using the Microsoft Windows XP operating system. Stimuli were presented on a 19″ LCD monitor (E1902S, Iiyama; 1024 × 768 pixels, 75 Hz refresh rate) and subtended a visual angle of about 20.8° × 28.1°.

All physiological signals were acquired and recorded using an integrated system (MP 150 system; BIOPAC Systems Inc., Goleta, CA, USA) and software package (AcqKnowledge 4.1; BIOPAC Systems Inc., Goleta, CA, USA) controlled by a PC (Latitude E5400; DELL). All signals were amplified, filtered, and digitized using a suitable BIOPAC amplifier (GSR100C, EMG100C, and ECG100C; BIOPAC Systems Inc., Goleta, CA, USA).

Skin conductance was measured with a pair of Ag/AgCl electrodes filled with 0.05 M sodium chloride gel placed on the volar surface of the distal phalanges of the first and second fingers of the left hand. Skin conductance signals were bandpass filtered at 0.05–10 Hz to control for drift.

Facial EMG activity was measured using Ag/AgCl electrodes with 4 mm diameter Ag/AgCl detection surfaces placed over the corrugator supercilii and zygomatic major muscle regions using the placement recommended by Fridlund and Cacioppo (1986). The raw EMG signals were bandpass filtered at 10–5000 Hz and digitized at a sampling rate of 10 kHz. The signals were then further bandpass filtered between 90 and 1000 Hz off-line and rectified and smoothed with a time-constant of 200 ms.

Electrocardiograms (ECGs) were recorded using the standard 3-lead montage (Eithoven lead 2 configuration). The ECG signals were bandpass filtered between 0.5 and 35 Hz and digitized at a sampling rate of 1000 Hz. Heart rates, in beats per minute, were calculated from the R–R intervals using an algorithm in AcqKnowledge 4.1. Interbeat intervals were checked in a tachograph and corrected if the R-wave triggers were misplaced.

### Procedure

Experiments were conducted individually in an electronically shielded and sound-attenuated room. Upon arrival, participants were told that the electrodes were harmless and that they could withdraw from the experiment at any time. All participants completed an informed consent form and successfully participated in the experiment. After all sensors were attached, task instructions were provided.

To habituate participants to the room and the equipment and assess baseline heart rate values in a resting period, ECGs were measured for 5 min while the participants rested. Participants were asked to relax in a chair but not to close their eyes to avoid falling into sleep. The heart rate data have been reported in Fujimura and Okanoya (2012). After the resting period, facial EMG, skin conductance, and ECG data were recorded during the picture-viewing task. A trial began with a 2 s fixation point followed by two successive pictures presented for 6 s each. After a 6 s blank screen, participants rated their feelings when viewing the last picture in the trial using the 9 × 9 Affect Grid, which assessed affect along the dimensions of valence and arousal (Russell et al., 1989). The participants indicated a square on a two-dimensional emotional space using a computer mouse. The rating screen was displayed until the participant responded. The inter-trial interval was 6 s.

### Data analysis

For EMGs and heart rates, the data were averaged over 6 s periods after the onset of the pre-target and target pictures. The change scores were calculated by subtracting the averaged values in the 1 s before pre-target picture presentation (i.e., baseline) from each averaged value. SCR magnitude was scored as the maximum value observed between 1 and 4 s after picture onset. SCRs are superimposed on a tonic level, which fluctuates with much greater magnitude than an acute response (Fowles et al., 1981). To clarify event-related SCRs to each stimulus, the baselines for the pre-target and target were set as the average values in the 1 s preceding pre-target and target picture onset, respectively. To normalize the distribution of the responses, the data were log transformed [log (SCR + 1)]. Physiological data were averaged within subjects and conditions. Three-way repeated-measures ANOVAs with the independent variables of pre-target emotion (unpleasant, pleasant, neutral), target emotion (unpleasant, pleasant), and period (pre-target, target) were conducted for the dependent variables of SCR, corrugator EMG, zygomatic EMG, and heart rate.

For the psychological ratings, valence and arousal ratings on a 9-point scale were collected for each trial. The averaged data across conditions were analyzed using a two-way repeated-measures ANOVA with pre-target emotion (unpleasant, pleasant, neutral) and target emotion (unpleasant, pleasant) as independent variables. Effect sizes are reported as $\eta_{p}^{2}$ (partial eta squared). The Greenhouse and Geisser (1959) adjustment was applied when needed, and corrected *p* and epsilon values are reported along with uncorrected degrees of freedom. Multiple comparisons were conducted with Tukey’s HSD.

## Results

### Subjective ratings

**Figure 2:**
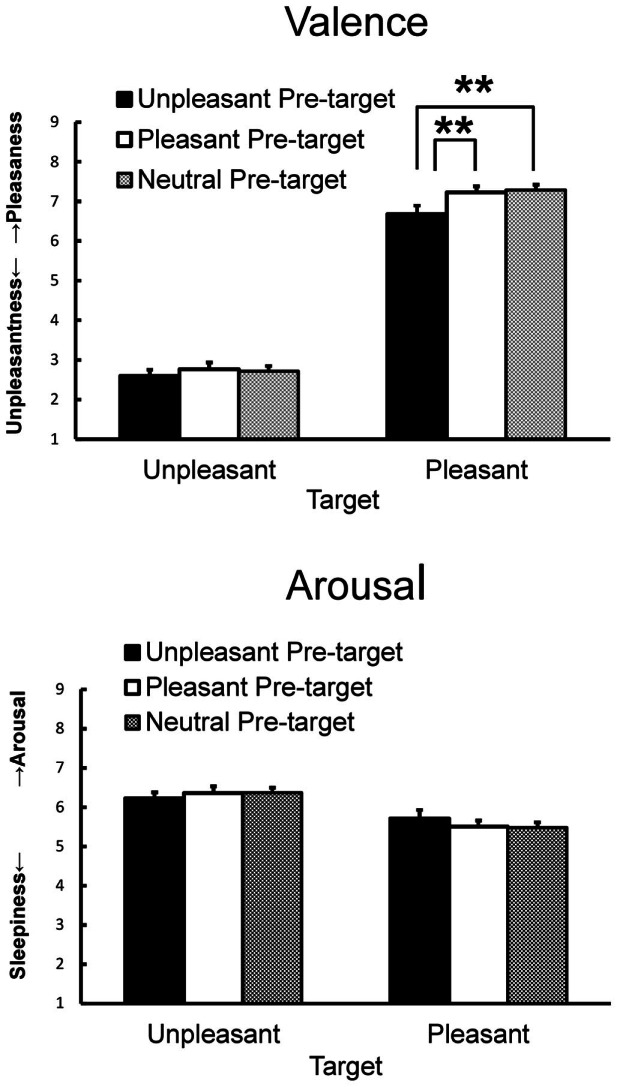
**Mean subjective valence and arousal ratings with SEMs**. Means with asterisks are significantly different at the *p* < 0.01 level for pre-target and target interactions.

Subjective ratings of valence and arousal when viewing the target pictures preceded by pleasant, unpleasant, and neutral pictures are shown in Figure 2. For valence ratings, ANOVA yielded significant main effects of pre-target emotion [*F*(2, 84) = 10.18, *p* < 0.01, $\eta_{p}^{2}$ = 0.20, ε = 0.77] and target emotion [*F*(1, 42) = 295.60, *p* < 0.01, $\eta_{p}^{2}$ = 0.88] and a significant interaction [*F*(2, 84) = 3.21, *p* < 0.05, $\eta_{p}^{2}$ = 0.07]. A simple main effect test revealed that the pleasant target picture preceded by the unpleasant picture elicited less pleasant feelings than when it was preceded by the pleasant or the neutral picture [*F*(2, 84) = 8.39, *p* < 0.01, $\eta_{p}^{2}$ = 0.17, HSD = 0.34, α = 0.05]. For arousal ratings, ANOVA yielded a significant main effect of target emotion [*F*(1, 42) = 11.17, *p* < 0.01, $\eta_{p}^{2}$ = 0.21] that was qualified by a significant interaction [*F*(2, 84) = 3.19, *p* < 0.05, $\eta_{p}^{2}$ = 0.07], indicating that the unpleasant target picture elicited more arousal than the pleasant target picture regardless of the pre-target picture.

### Skin conductance

**Figure 3:**
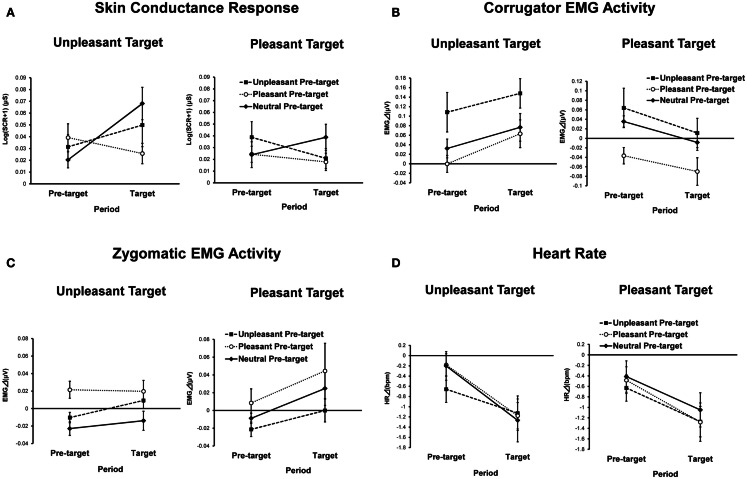
**Means and SEMs of physiological indices as a function of pre-target emotion, target emotion, and period: (A) skin conductance response; (B) corrugator EMG activity; (C) zygomatic EMG activity; (D) heart rate**.

Skin conductance magnitudes as functions of conditions and periods are presented in Figure 3A. ANOVA revealed a significant main effect of target emotion [*F*(1, 42) = 5.04, *p* < 0.01, $\eta_{p}^{2}$ = 0.11], indicating that SCRs to the unpleasant target picture were significantly greater than those for the pleasant target picture. The pre-target emotion and period interaction was also significant [*F*(2, 84) = 5.07, *p* < 0.05, $\eta_{p}^{2}$ = 0.11, ε = 0.81]. A simple main effect test revealed that SCRs to the target pictures preceded by pleasant pictures were significantly reduced compared to SCRs to target pictures preceded by neutral pictures [*F*(2, 84) = 4.46, *p* < 0.05, $\eta_{p}^{2}$ = 0.10, HSD = 0.02, α = 0.05]. Additionally, SCRs during the target period were greater than those during the pre-target period when the pre-target picture was neutral [*F*(1, 42) = 5.12, *p* < 0.05, $\eta_{p}^{2}$ = 0.11].

### Corrugator EMG

Corrugator EMG activities as a function of condition and period are presented in Figure 3B. ANOVA yielded a significant main effect of target emotion [*F*(1, 39) = 12.03, *p* < 0.01, $\eta_{p}^{2}$ = 0.24] that was qualified by a significant interaction between target emotion and period [*F*(1, 39) = 9.47, *p* < 0.05, $\eta_{p}^{2}$ = 0.20]. A simple main effect test showed that unpleasant target pictures elicited greater corrugator EMG activities than pleasant target pictures [*F*(1, 39) = 13.72, *p* < 0.01, $\eta_{p}^{2}$ = 0.26]. Additionally, a simple main effect of period was significant only for the pleasant target picture condition [*F*(1,39) = 14.65, *p* < 0.01, $\eta_{p}^{2}$ = 0.27], indicating that corrugator EMG activities in response to the pre-target pictures were decreased by viewing of the pleasant target pictures.

The main effect of pre-target emotion was significant [*F*(2, 78) = 11.05, *p* < 0.01, $\eta_{p}^{2}$ = 0.22, ε = 0.80], indicating that the corrugator EMG activities during trials with an unpleasant pre-target picture were significantly greater than those of trials with pleasant or neutral pre-target pictures (HSD = 0.04, α = 0.05).

### Zygomatic EMG

Zygomatic EMG activities as a function of condition and period are presented in Figure 3C. ANOVA yielded a significant main effect of pre-target emotion [*F*(2, 78) = 5.15, *p* < 0.05, $\eta_{p}^{2}$ = 0.12, ε = 0.71], indicating that zygomatic EMG activities during trials in which the pre-target picture was pleasant were higher than those in trials in which the pre-target picture was unpleasant or neutral (HSD = 0.02, α = 0.05). The main effect of period was also significant [*F*(1, 39) = 5.98, *p* < 0.05, $\eta_{p}^{2}$ = 0.13], showing that zygomatic EMG activities during the target period were stronger than those during the pre-target period.

### Heart rate

Figure 3D shows heart rate changes for each condition and period. ANOVA revealed a significant main effect of period [*F*(1, 39) = 27.69, *p* < 0.01, $\eta_{p}^{2}$ = 0.42], indicating that heart rate deceleration during target-picture viewing was significantly greater than during pre-target picture viewing. There were no other main effects or interactions.

## Discussion

The present study found that psychological, autonomic, and facial responses to emotional stimuli were modulated by the temporal context of preceding emotional stimuli. First, the subjective feelings induced by the unpleasant target picture were carried over into the subjective feelings induced by the subsequent pleasant pictures; this finding is consistent with the results of Waugh et al. (2011). Importantly, we clarified the existence of a carry-over effect for unpleasant feelings that was not present for pleasant feelings, as the pleasant pre-target pictures did not enhance pleasant feelings during pleasant-picture viewing. These results suggest that unpleasant feelings last longer than pleasant feelings and are more able to contaminate subsequent pleasant feelings. This positive-negative asymmetry in the carry-over effect might be due to a negativity bias; negative emotions, compared to positive emotions, have a greater impact on psychological phenomenon (Cacioppo et al., 1994; Ito et al., 1998; Baumeister et al., 2001). Negativity bias has been confirmed in several domains, including the evaluative categorization task (Ito et al., 1998), impression formation (Ikegami, 1993), and decision-making (Katahira et al., 2011). In the present study, it is possible that unpleasant feelings informed participants that the environment was threatening, which led to subsequent inhibition of activation of the appetitive system when viewing pleasant pictures. Consequently, prolonged negative emotions might alert organisms to cope with subsequent emotional events. This finding has important implications for emotional disorders. For example, anxiety disorders characterized by hypervigilance toward potential threats and excessive worry are similar to the states of the subjects during presentation of unpleasant pre-target stimuli in emotional contexts. Accordingly, patients with anxiety disorder might feel less pleasantness due to hypervigilance caused by pre-existing unpleasant feelings.

Regarding autonomic responses, the SCRs to unpleasant target stimuli were greater than those to pleasant target stimuli. As the unpleasant target pictures elicited greater arousal that did the pleasant target stimuli, this result is in line with previous findings that SCRs while viewing emotionally laden stimuli reflect arousal levels due to the stimuli (Lang et al., 1993). More importantly, the SCRs to emotional target stimuli were smaller when preceded by pleasant stimuli than when preceded by neutral stimuli. This finding suggests that viewing pleasant pictures decreases the intensity of the motivational activation elicited by subsequent emotional stimuli. From the feelings-as-information perspective (Schwarz and Clore, 1983, 2007), it is possible that pleasant feelings induced by viewing pre-target pleasant pictures signals benign environments, which leads to underestimation of the perceived arousal level of the target stimuli. Hence, the target stimuli might elicit less arousal when preceded by moderately arousing pleasant stimuli, which would result decreases in the amplitudes of the SCRs. Although neutral stimuli can also signal benign environments, pre-viewing neutral pictures elicited no significant “feelings” that were sustained during the presentation of the target stimuli. Thus, SCRs to the emotional target stimuli did not decrease when preceded by neutral pre-target stimuli as much as they did when preceded by pleasant pre-target stimuli.

In contrast, corrugator activity in response to the target stimuli was not modulated by the pre-target stimuli. Specifically, this result indicates that, regardless of the hedonic valence of the pre-target stimuli, corrugator responses to the unpleasant target stimuli were greater than those to the pleasant target stimuli. Importantly, we found that corrugator activity in response to the pre-target stimuli was significantly decreased when pleasant target stimuli were presented, suggesting that viewing pleasant pictures inhibited or undid facial corrugator activity. This result can be explained by the undoing effect (Fredrickson and Levenson, 1998; Mancuso et al., 2000), wherein positive emotions facilitate recovery from the cardiovascular activation induced by negative emotions. In the current study, the pleasant feelings induced by viewing pleasant pictures might undo the enhanced facial corrugator activity during pre-target picture viewing. These findings represent new evidence for the undoing effect of positive emotion. Given that facial expressions are visible emotional responses, sustained negative expressions during positive events would prevent people from constructing good relationships with others. Therefore, it is reasonable that negative expressions return to neutral immediately after facing pleasant stimuli. However, unlike the result of Waugh et al. (2011), our findings indicate that corrugator responses to unpleasant target stimuli were not enhanced, even when preceded by unpleasant stimuli. This discrepancy may be due to differences in presentation conditions. Because Waugh et al. (2011) presented three successive emotional pictures with the same valence, unpleasant feelings may have been strongly induced by viewing unpleasant pictures and subsequently carried over to facial responses to the next unpleasant picture.

Zygomatic activity showed no effect of contextual modulation. However, zygomatic activity was enhanced when viewing pleasant pictures during the pre-target period, suggesting that the set of pleasant pictures used in this study had positive properties. Additionally, zygomatic activity during the target period was greater than that during the pre-target period regardless of the valence properties of the target stimuli. This finding is due to the quadratic correlation between valence and zygomatic response; i.e., zygomatic activity is enhanced by both positive and negative valences (Lang et al., 1993). This result suggests that zygomatic activity is facilitated by pleasant and unpleasant stimuli alike.

Heart rate was also not contextually modulated. Heart rate deceleration was prominent during the target period regardless of the valence properties of the pre-target stimuli. As heart rate deceleration indicates orienting responses to incoming stimuli (Sokolov, 1963; Lacy and Lacy, 1970), orienting responses were not modulated by emotional context.

The effects of contextual modulation on emotional responses differed as measured by psychological and physiological responses. Specifically, unpleasant feelings signaled threatening environments that resulted in the attenuation of pleasant feelings induced by target stimuli, whereas pleasant feelings signaled benign environments and resulted in decreases in the amplitude of SCRs to the target stimuli. One possible explanation for this discrepancy might be the difference as a function of emotional response. SCRs reflect activation of the sympathetic system to prepare for actions, fight, or flight (Lang et al., 1997). Thus, large SCRs induce a high metabolic cost. When unthreatening situations are signaled by pleasant stimuli, it is possible that SCRs to a subsequent emotional stimulus are inhibited to reduce energy consumption. On the other hand, subjective feelings impose less cost on organisms compared to autonomic responses. Additionally, subjective feelings are long-lasting and affect cognition and behavior (Dreisbach and Goschke, 2004; Clore and Huntsinger, 2007). Thus, prolonged subjective negative feelings might play a role in the control of cognition and behavior in preparation for any contingency in the emotional context. Furthermore, only facial corrugator activity showed an influence of pleasant target picture viewing on the response to the pre-target picture. The temporal properties of the occurrence of facial muscle activity could explain this result. Facial muscle activity occurs rapidly within 500 ms after the onset of pictures (Dimberg and Thunberg, 1998) and is maintained after the disappearance of the pictures (Weyers et al., 2006). Viewing a pleasant target picture may have rapidly decreased the lasting corrugator activity in response to the pre-target pictures. Further research is needed to reveal the influence of the temporal properties of physiological and psychological measures.

In sum, the present study showed several types of contextual modulation of emotional responses regarding subjective feelings, SCRs, and corrugator activities. Pre-existing feelings influence how we react to subsequent emotional stimuli. Hence, the motivational significance of the emotional stimuli is altered, resulting in contextual modulation of responses to emotional stimuli. These findings provide beneficial evidence for the understanding of psychological and physiological responses to successive emotional events.

## Conflict of Interest Statement

The authors declare that the research was conducted in the absence of any commercial or financial relationships that could be construed as a potential conflict of interest.

## Footnotes

^1^The IAPS pictures numbers for the unpleasant pictures are 1271, 1301, 3230, 6231, 9041, 9295, 9419, 9421, 9530, 9610; for the pleasant pictures: 1440, 1710, 2156, 2347, 4614, 5199, 5833, 5910, 7502, 8190; for the neutral pictures: 2411, 5740, 7004, 7217, and 7491. These pictures did not include any highly arousing pictures (e.g., mutilation as unpleasant or sexual as pleasant pictures)
